# The Complement System: A Potential Therapeutic Target in Liver Cancer

**DOI:** 10.3390/life12101532

**Published:** 2022-09-30

**Authors:** Meng Yuan, Li Liu, Chenlin Wang, Yan Zhang, Jiandong Zhang

**Affiliations:** 1 School of Clinical Medicine, Weifang Medical University, Weifang 261053, China; 2 Medical Integration and Practice Center, Cheeloo College of Medicine, Shandong University, Jinan 250100, China

**Keywords:** liver cancer, complement system, tumor microenvironment, caner stem cells, tumor therapy

## Abstract

Liver cancer is the sixth most common cancer and the fourth most fatal cancer in the world. Immunotherapy has already achieved modest results in the treatment of liver cancer. Meanwhile, the novel and optimal combinatorial strategies need further research. The complement system, which consists of mediators, receptors, cofactors and regulators, acts as the connection between innate and adaptive immunity. Recent studies demonstrate that complement system can influence tumor progression by regulating the tumor microenvironment, tumor cells, and cancer stem cells in liver cancer. Our review concentrates on the potential role of the complement system in cancer treatment, which is a promising strategy for killing tumor cells by the activation of complement components. Conclusions: Our review demonstrates that complement components and regulators might function as biomarkers and therapeutic targets for liver cancer diagnosis and treatment.

## 1. Overview of Liver Cancer

Liver cancer is the sixth most common cancer and the fourth most fatal cancer worldwide [1]. By 2025, the number of individuals influenced by liver cancer annually is estimated to reach more than 1 million [2]. In primary liver cancer, hepatocellular carcinoma (HCC) accounts for 80–90% of all cases, and cholangiocarcinoma (CCA) represents 10–15% [3]. The liver is the most common organ in metastasis from plenty of solid tumors. Patients with metastatic liver cancer have a poor 5-year survival rate and quality of life [4].

**Figure 1:**
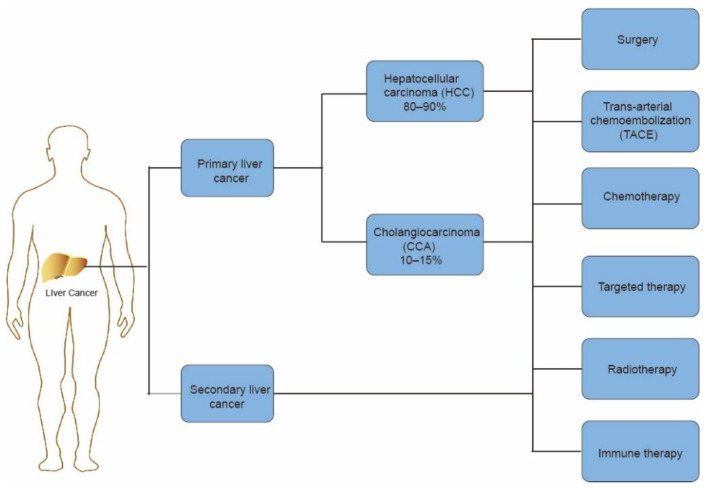
Introduction to liver cancer histological classification and therapeutic methods.

A majority of liver cancer patients are diagnosed at an advanced stage, resulting in poor prognosis. The current therapies include surgery, trans-arterial chemoembolization (TACE), chemotherapy, sorafenib-based targeted therapy, radiotherapy, and immune therapy [5] (Figure 1). In terms of advanced liver cancer, TACE and sorafenib are the main therapies. As for advanced liver cancer patients, sorafenib, a multi-target inhibitor of tyrosine kinases and c-RAF, acts as an effective treatment [6]. In the SHARP study (Sorafenib HCC Assessment Randomized Protocol), the use of sorafenib was able to increase median overall survival compared with a placebo, which shows the efficacy of sorafenib in HCC [7]. However, unfortunately, up to 60% of patients cannot benefit from the treatment and produce drug resistance [8]. The emergence of immunotherapy is a new strategy in the treatment of liver cancer. The liver is a unique immune organ with numerous types of immune cells [9]. In cancer immunotherapy, blocking immune checkpoints can increase the T-cell-mediated antitumor immune response by disrupting the signal pathways to attack cancer cells [10]. Such immunotherapies consist of anti-PD-1/PD-L1, anti-CTLA-4 inhibitors, and others, which have already achieved initial results in the treatment of liver cancer [11]. On the basis of these developments, there are significant improvements in patients’ quality of life and overall survival rates [12]. 

**Figure 2:**
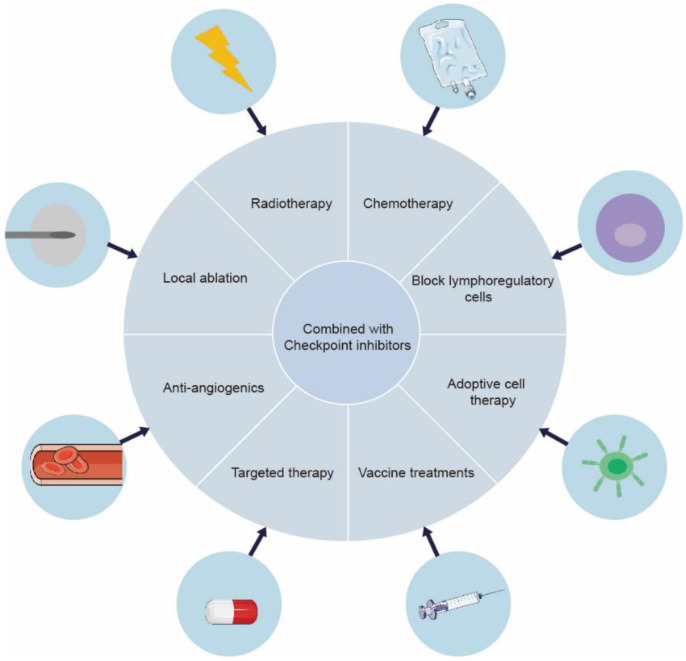
Expanding the synergistic effect of ICIs in combination with other strategies in liver cancer. A series of clinical and preclinical investigations have shown that ICI-based combination therapy significantly improves therapeutic efficacy. Therefore, the novel and optimal combinatorial strategies need further research.

However, there still exists resistance towards immune checkpoint inhibition in a majority of cancer patients because of immunogenic mutations in cancer, reduced T cell infiltration, etc. [13,14]. Whereas current cancer immunotherapy primarily employs cytotoxic T cells, effectors of the adaptive immune system, other factors certainly influence the cancer therapeutic modalities. Chronic inflammation from the patient’s underlying liver disease damages the immune response. More than 90% of HCCs emerge in the setting of chronic liver disease, such as liver cirrhosis and chronic hepatitis, which is usually caused by hepatitis B or hepatitis C infection [15]. In this manner, cancer cells escape immune surveillance by creating an immunosuppressive environment [16]. It has been suggested that the activation of the NF-κB and JAK-STAT pathways is correlated with HCC-related key inflammatory signaling pathways [17]. In a randomized clinical trial (IMbrave050), it was demonstrated that a multimodal approach enhances liver cancer immunotherapy efficacy compared with monotherapy [18]. Radiotherapy and chemotherapy could induce cell death, releasing tumor antigens and activating antigen presenting cells (APCs), further contributing to anti-tumor immunity by the presentation of tumor antigens to effector cells [19]. Combining checkpoint suppression with other current treatments for liver cancer can produce a synergistic effect [20]. For advanced HCC patents, atezolizumab plus bevacizumab has been approved as the first-line treatment standard in 2020, based on a global, open-label, phase III trial (IMbrave150) [21,22]. Combination anti-PD-1/PD-L1 antibodies and anti-angiogenic Tyrosine kinase inhibitors (TKIs) showed synergistic effects in the treatment of HCC, which were related to the inhibition of VEGF [23]. In tumor vasculature, the inhabitation of VEGF signaling contributes to the formation of vessel pruning and vessel normalization. In addition, VEGF acts as an effective immunomodulatory factor, directly influencing myeloid-derived suppressor cells (MDSCs), regulatory T Cells (Treg cells), and effector T cells, which enhance tumor immunosurveillance capabilities [23,24]. Cellular-based immunotherapy has also shown great advantages in primary liver cancer. Vaccine treatments, active and passive cellular immunotherapy, and myeloid cell-based immunotherapy are the positive areas of investigation [25] (Figure 2).

In many solid tumors, regulation of the complement system can be advocated as a potential immunotherapy tool [26,27,28]. Complement plays a role in both the removal of foreign objects and the inflammatory response of the innate immune system [29]. In addition, activation of complement components can heighten adaptive immune responses and polarize immune responses to cause tumor development and metastatic spread [30]. Complement proteins are primarily produced in the blood, but they are also synthesized by a selection of other cell types, including macrophages, tumor cells, etc. [31]; they can play a major role in a variety of non-immune-related processes as well [30]. Consequently, we expect to clarify the role of the complement system in liver cancer in this review, especially centering on its mechanism in the occurrence, development, and transfer of liver cancer. We further explore the possibility of using complement modulators in the treatment of liver cancer.

## 2. Introduction to the Complement System

### 2.1. Activation of the Complement System

The complement system can be activated by three distinct pathways: the classical pathway (CP), the lectin pathway (LP), and the alternative pathway (AP). These pathways all generate the anaphylatoxins C5a and C3a to form membrane attack complex (MAC) via formatting C3 convertase [32]. C1 protein complex includes C1q, C1r, and C1s. Cell death protein C1q binds to apoptotic cell membranes and is recognized by phagocytes [33]. The CP activation occurs when IgG or IgM antibodies, complexed with antigens, bind to the C1 complex, which triggers C1r and C1s in the C1 protein complex [34]. When activated C1s hydrolyze C4, C4a and C4b are formed, whereas C2 is transformed into C2a and C2b [35]. A C3 convertase is formed, namely C4bC2a. Both CP and LP produce the same C3 convertase, whereas the alternative pathway produces a different C3 convertase [36]. AP activation leads to the hydrolysis of C3 into bioactive C3(H2O), which binds to factor B and is recognized by factor D, forming the alternative C3 convertase: C3(H2O)Bb [37]. C3 convertase can cleave C3 into C3a and C3b, which combine with C3 convertase to form C5 convertase. C5a and C5b fragments are formed when C5 is cleaved by convertase [38]. Subsequently, the large C5b fragment binds to C6, C7, C8, and C9 to assemble MAC and cause target lysis, calcium influx, and cell apoptosis [32]. MAC initiates the cell cycle by generating calcium influx, resulting in the process of tumorigenesis [39]. Likewise, crucial proteins in apoptosis could be inhibited by MAC. C3a and C5a act as available anaphylatoxins by combining with their G-protein-coupled receptors—C3aR and C5aR1 [40]. In addition, there exists another C5a receptor, C5aR1, that takes part in C5a responses, which is not a G-protein-coupled receptor [41]. Our understanding of the function of C5aR2 is limited; evidence suggests that C5aR1 acts on the cell surface and may be interfered with by C5aR2 [41]. 

### 2.2. Regulation of the Complement System

**Figure 3:**
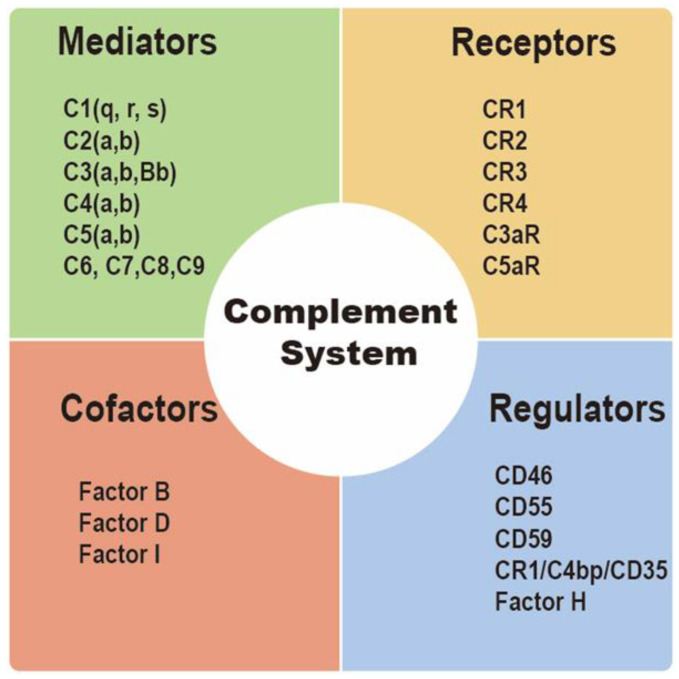
Introduction to complement components. The complement system consists of mediators, receptors, cofactors, and regulators.

Integral membrane-bound proteins as well as plasma proteins influence complement activation [42]. Complement regulatory proteins (CRPs), including membrane cofactor protein (CD46), decay-accelerating factor (CD55), protectin (CD59), complement receptor type 1 (CR1/C4bp/CD35), and factor H, are membrane-bound proteins that prevent complement-mediated cytolysis [43]. Factor-H can regulate the formation of C3a convertase via competitively binding to C3b [32]. On the other hand, CD55 also generates the decay of the CP, AP, and C3 and C5 convertases [44]. CR1/CD35 and CD46 might act as cofactors of factor I in the formation of C3b and C4b to regulate T cell function [45]. Moreover, CD59 may play a role in formation of MAC by inhibiting C9 insertion into the lipid bilayer [46]. MAC acts a transmembrane pore complex, inducing increases in membrane permeability and membrane destabilization, and finally resulting in osmotic lysis of the target cell, metabolically inert targets, and certain bacteria (Gram-negative only) [47] (Figure 3). 

### 2.3. The Immunomodulatory Role of the Complement System

The complement system plays a role in the immune system and represents the link between innate and adaptive immunity. C1q enhances apoptotic-cell and immune-complexed clearance to regulate T cell metabolism [48]. Meanwhile, the effect is always accompanied by IL-4 and IL-10. In circulation, tissues, and intracellular processes, the complement system could be activated by an unconventional, convertase-independent pathway [47]. In addition, tumor cells could release (pro)cathepsin L to cleave C3 [49]. Haoran Zha found that it was C3a emerging from mouse tumor cells, not C3a generated exogenously, that drove TAM polarization, contributing to an immunosuppressive tumor microenvironment (TME) [50]. Upon antigen presentation, the differentiation of helper T (TH) cells into Th1, Th2, Th17, or Tregs and the transmission of co-stimulation and antigen presentation signals were tightly regulated by the combination of C3a/C5a and C3aR/C5aR1 in APC and T cells [51,52]. The activation of C3aR and C5aR1 affected FOXP3 genes of Tregs and downregulated their immunosuppressant activity [53]. Some findings demonstrated that C5a could promote the differentiation of Tregs in a dose-dependent manner. High concentrations of C5a played a positive role in the Treg differentiation process [54].

Some studies have demonstrated that tumor cells can avoid potential self-harm by expressing CRPs [55]. As everyone knows, complement proteins are mainly produced in the liver. Complement proteins, locally generated by T cells and APCs, can exert an important role in immunomodulation [56]. Human complement receptor 2 (CR2/CD21) is naturally expressed predominantly on the surface of B cells and follicular dendritic cells. C3d is a ligand for CR2. The interaction of C3d and CR2 could promote antigen presentation in the process of germinal-center B cells differentiating into memory B cells [57]. CD59, a complement regulatory protein that downregulates complement-mediated cell lysis, could mediate immune regulation and produce resistance to cancer cells [58]. Alterations in CD59 always followed induction of tumorigenesis or cancer progression. Recent studies have shown that significantly lower CD59 correlates with poor survival in breast cancer, while high expression of CD59 protein induced decreased overall survival rates in colorectal cancer, prostate cancer, etc. [59,60,61]. 

### 2.4. Prospects for the Role of the Complement System in Cancer

Activating the complement system assists the body in defending itself against invaders, repairing damaged tissue, and maintaining homeostasis [62]. The activation of anaphylatoxins is known to cause vasodilation, increased vasopermeability, and neutrophil chemotaxis by causing degranulation of macrophages, neutrophils, and mast cells and production of cytokines [63]. Interactions between complement components also coordinate innate immune responses with adaptive immune responses [64]. Complement dysfunction leads to autologous damage and immune disorders, and the complement system has been implicated in the pathogenesis of a wide range of diseases [64]. Atypical hemolytic uremic syndrome, systemic lupus erythematosus, and C3 glomerulopathies are closely associated with inadequate activation of complement [65]. Recently, seminal discoveries have shown that the complement system is closely related to tumor progression.

**Figure 4:**
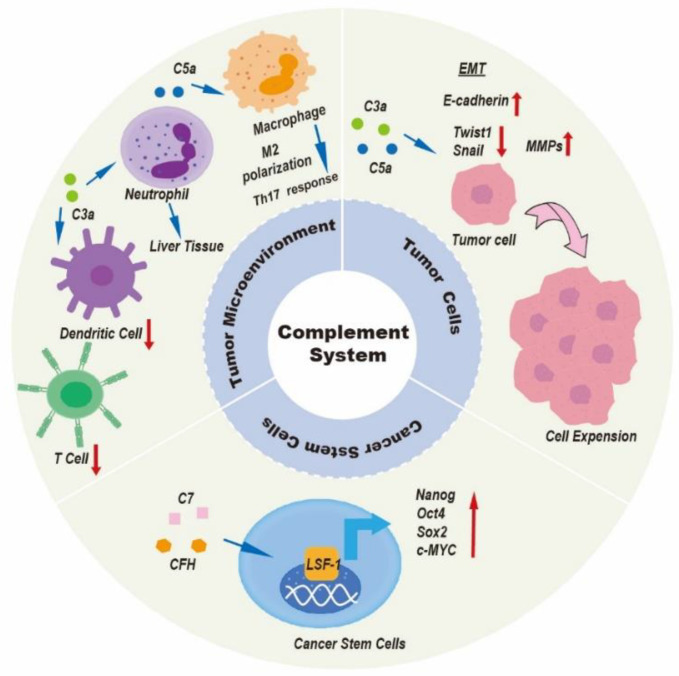
Introduction to role of the complement system in liver cancer. Complement components participate in several biological processes involved in liver cancer progression by regulating the tumor microenvironment, tumor cells, and cancer stem cells. The complement system can modulate immunosuppression within the tumor microenvironment; contribute to the establishment of the premetastatic niche; accelerate tumor invasion and growth; induce extracellular angiogenesis; and induce cancer stem cells to exert their pro-tumor properties.

Disorders of the complement system, either from overactivation or as a result of reduced complement levels, may contribute to tumor initiation and tumor progression [66]. The complement system is an important part of the body’s immune system, which plays a key role in both innate and adaptive immune responses. Innate immunity is the cornerstone of initiating anti-tumor immune effects. Tumor cells are first recognized by the innate immune system as “non-self” substances, which triggers anti-tumor cytotoxicity to initiate the killing of tumor cells. The complement system plays a double role in tumor progression. Although the function of the complement system has been inconsistently described in several papers, most studies point to the pro-tumor activation of complement by tumors. In fact, because of different immune microenvironments in different types of cancer, the complement system has a variety of modes of action, which are largely influenced by the characteristics of the tumor cells. In the following sections, we will focus on the roles of elements of the complement system in tumor biology and their potential clinical applications in liver cancer. Figure 4 and Table 1 summarize the function of the complement system in liver cancer in some studies.

## 3. Role of the Complement System in Liver Cancer

### 3.1. Role of the Complement System in the TME

The survival of tumor cells despite immune attacks plays an important role in tumor progression [67]. Almost all studies indicate that complement activation play a critical role in tumor immunosuppression [68]. The G-protein-coupled receptors, C3aR and C5aR, present on most immune cells allow C3a and C5a to carry out their functions [47]. Complement C5a is linked to poorer cancer prognosis and advanced tumor stage in HCC. Complement C5a activation can motivate Th-17 responses in TAMs by inducing Th-17-related cytokines and their regulatory genes [69]. TAMs, the primary cell type in TME, can be polarized to M1 (anti-tumorigenic) or M2 (pro-tumorigenic) phenotypes [70]. In a mouse model of colon cancer, C5a interacted with C5aR1 to promote M2 polarization of TAMs, resulting in hepatic metastasis of colonic carcinoma [71]. In contrast, the influence of complement on M1-like macrophages has not been clarified. The Th-17 T-cell response could release cytokines (IL-17, IL-21, and IL-22) to promote liver tumor progress; several studies have shown that this was correlated with poor clinical outcome in HCC patients [69]. In addition, C5aR1 could promote the expression and secretion of interleukin-6 (IL-6) in hepatoma cells [72]. IL-6 is a multipotent cytokine that causes inflammation through JAK-STAT3, promotes tumor progression by regulating the cell cycle and avoiding apoptosis, and induces cancer drug resistance [73]. As a result, blocking the activation of complement components in cancer could contribute to enhancing the efficacy of tumor immunotherapy [74].

Anaphylatoxins C5a and C3a serve as a potent chemoattractant for neutrophils [75]. TANs have been associated with cancer progression, where it has been shown that the complement system may generate TAN chemotaxis in malignancies [76]. How does complement signaling promote tumor progression via influencing tumor-associated neutrophils (TANs)? Neutrophil phenotypes in the TME can be described as high-density neutrophils (HDNs) and low-density neutrophils (LDNs) [77]. Immature low-density neutrophils (iLDNs) have been found to promote liver metastasis differently from HDNs [78]. First, Brian E. Hsu and colleagues observed that the expression of C3aR on the surface of iLDNs was increased [79]. Second, the C3a/C3aR signaling axis resulted in iLDNs accumulating in liver metastases of breast cancer [79]. In addition, C3a/C3aR signaling was interrupted, both in vivo and vitro, thus eliminating the chemotactic reaction of iLDNs [79]. More importantly, iLDNs primarily gather in liver tissue instead of lung tissue, leading to the formation of liver metastases due to this novel mechanism [79]. Therefore, inhibition of liver metastasis through suppression iLDN chemotaxis in response to C3a/C3aR signaling reveals a novel mechanism.

Complement plays an important role in tumor metastasis by causing a change in metastasis niche [80]. The premetastatic niche is an advantageous microenvironment for tumor growth, affecting angiogenesis, the formation of extracellular matrix, inflammation, and tumor immune escape [81]. It can promote the implantation of tumor cells in organs that are distant from the primary tumor site [82]. In distant organs, different cancer types selectively change the microenvironment niche of these target organs. We have found that C3aR can contribute to secondary hepatic malignancy by promoting the aggregation of iLDNs to liver metastases [79]. The formation of an immunosuppressive microenvironment is one of the important reasons for a pre-metastatic niche. Surya Kumari and colleagues also found that C5aR could influence the recruitment of MDSCs to tumor metastasis. In this metastatic syngeneic murine model of breast cancer, the inhibition of C5aR did not affect the occurrence of primary tumors, and a C5aR signal generated the formation of liver and lung metastases. Previous studies have shown that inhibition of C5aR can block the invasion of immunosuppressive cells into the liver and lungs in breast cancer [83]. However, the mechanism by which the complement receptors on the surface of immunosuppressed cells promote metastasis of their tumor cells and the mechanism by which the location of the tissue for metastasis is selected still need further study.

**Figure 5:**
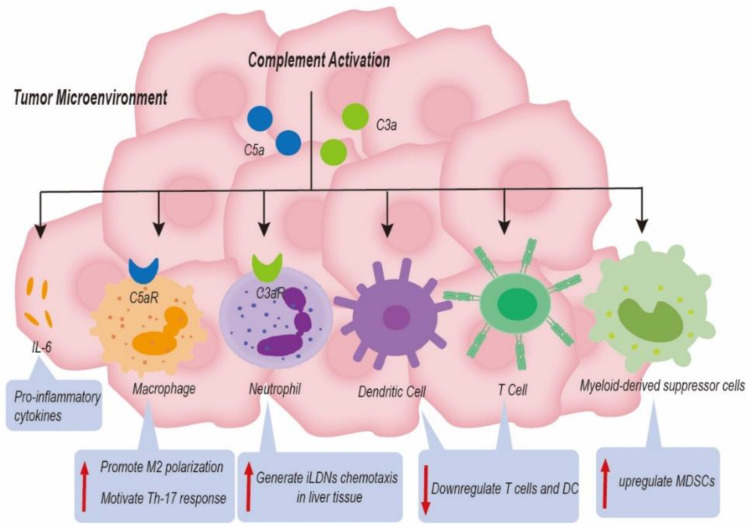
Complement-based mechanisms associated with liver cancer progression. Complement components induced the formation of an immunosuppressive status, a change in metastasis niche, and the release of proinflammatory cytokines to promote tumor progression by upregulating immune suppressing cells and by downregulating immune promoting cells in the tumor microenvironment.

Several studies have shown that complement C3 served as an immune modulator to enhance liver cancer progression through downregulation of T cells and dendritic cells (DC) and upregulation of tumor-related MDSCs [84,85]. The key immune suppressive cells, such as MDSCs and Tregs, assist cancer cells in escaping immune surveillance. Interestingly, HCC patients with high expression of C2 harbored a high level of CD4 T cells, while HCC patients with lower C2 expression harbored a higher proportion of macrophage M0 cells [86]. The complement system not only serves as an innate immune modulator but also modulates secondary immune cell activities. In plenty of solid tumors, activation of the complement system also played a critical role in regulating the function of tumor infiltrating lymphocytes (TILs) in TME [87,88]. Nevertheless, there is still a need to carry out intensive studies on its role in liver cancer (Figure 5).

### 3.2. Role of the Complement System in Tumor Cells

Besides its role of regulating immunity, the complement system also takes part in a number of non-immune-related oncogenic processes. Autocrine activation of C5aR1 promoted the invasion and migration of HCC cells and was highly correlated with capsular infiltration, tumor stage, and epithelial–mesenchymal transition (EMT)-related indicators [89,90]. In HCC cells, C5a/C5aR1 signal enhanced EMT by upregulating Snail expression and upregulating Claudin-1 and E-cadherin expression were also related to activation of ERK1/2 [89]. Similarly, locally produced C3a binding to C3aR on HCC cells performed the same function [91].

Circulating tumor cells are referred to as CTCs, which are tumor cells that enter human peripheral blood. CTCs are derived from the primary tumor tissue and circulate freely in the patient’s bloodstream. They could act as the origin of hematogenous cancer metastasis with EMT transition properties [92]. The HCC patient with high expression of C5aR1 would be vulnerable to developing vascular invasion. The C5a/C5aR1 axis is capable of maintaining mesenchymal phenotypes and promoting the dissemination of CTCs in distal organs via the upregulation of INHBA/Activin and the phosphorylation of smad2/3 [93]. Besides EMT transition properties, in the original step of cancer metastasis, tumor cells secrete stromelysins and matrix metalloproteinases (MMPs), inducing the loss of cell–cell adhesion and an improvement in their motility [94]. In hepatobiliary duct malignancy, C5aR1 enhanced the expression of MMPs to increase cell locomotion and promote skeleton rearrangement [95].

Similarly, C5aR1 was found to correlate with liver metastasis of gastric cancer [96]. Interestingly, C5aR1 was highly expressed in hepatitis B virus (HBV)-related HCC cells. The uppermost cause of HCC in the world is still chronic infection with HBV [97]. Furthermore, HBV and HCV infection are susceptible to the recurrence of HCC. The protein HBc could promote proliferation of HCC cells by signaling through C5aR1. The activation of C5aR1 stimulated JNK and ERK signal pathways [72]. Bendong Chen et al. have demonstrated that C3aR/C5aR1 generated arrest of the cell cycle G0/G1 phase and apoptosis in HCC cells [90]; it also downregulated the expression of PCNA and Ki-67 [90]. Such multiple mechanisms can affect tumor cells directly to inhibit tumor growth or promote apoptosis. Complement factor H-related 3 (CFR3) is overexpressed in liver tissue rather than other tissues [98]. Based on that fact, we suspected that CFR3 was related to liver cancer. Just as we expected, there was a study that demonstrated that CFR3 could inhibit the PI3K/AKT/mTOR signal pathway to promote apoptosis and suppress proliferation of HCC cells [99]. C1q enhanced the expansion of hematopoietic progenitor cells (HPC), resulting in hepatic fibrosis and hepatocarcinogenesis [100]. C1q inhibition prevented cancer in patients with chronic hepatitis by effectively regulating β-Catenin pathway activation. Using a C1q inhibitor could prevent the occurrence and progression of liver cancer [100]. A more improved understanding of regulatory mechanisms has opened up novel approaches to liver cancer diagnosis, prognosis and therapy.

### 3.3. Role of the Complement System in Cancer Stem Cells

HCC is a cancer characterized by heterogeneity, which may be caused by cancer stem cells (CSCs), which are a subset of cells with characteristics of stem/progenitor cells [101,102]. They are a very small proportion of tumor cells, accounting for 1% in solid tumors [103]. CSCs can propel tumor occurrence, resistance, and relapses. They are not only influenced by signaling molecules in CSCs as well as non-CSCs but also by their plasticity. Therapeutics, such as chemo- and radiotherapy, and the tumor microenvironment can all influence CSCs [104]. CSCs are also known as cancer initiating cells (CICs). In varieties of recurrent and metastatic disease, CSCs are resistant to radiotherapy and chemotherapy [105,106]. Several studies have demonstrated the mechanism by which a huge number of biomarkers in CICs promotes resistance to tumor therapies [104].

Early investigation indicated that C7 (complement component 7) and CFH acted as significant markers on the surface of liver CICs. The up-expression of C7 and CFH can contribute to tumor cell growth and the formation of the tumorsphere [107]. Hyang Sook Seol and their colleagues discovered that C7 and CFH could stimulate the expression of LSF-1, which was located in the nucleus and bound to promoters of Nanog, Oct4, Sox2, and c-MYC genes; this lead to upregulation of the dryness factor, which ultimately increased the dryness of the cancer [107].

High levels of CFH mRNA were correlated with improved survival rates, while reduced CFH was associated with poor survival of HCC patients. CFH was involved in the activation of AP complement, the absence of which contributes to abnormal inflammation and thus results in hepatic carcinogenesis [108]. Recently chimeric antigen receptor (CAR)-T-cell approaches to targeting CD33 + CSCs have been shown to be hugely advantageous in liver cancer therapies. A novel clinical trial (NCT02541370) verified that CD133-directed CAR-T-cells therapies greatly promote median progression-free survival in HCC patients who have no response to sorafenib. Beyond that, targeting complement components to inhibit the expression of CSCs is also a good idea. However, the concrete mechanism of C7 and CFH promotion of the expression of LSF-1 is not clear. This finding provides a new way of reversing drug resistance in tumors.

## 4. Therapeutic Potential of Modulating Complement System in Liver Cancer

Over the last several years, only a limited number of studies have investigated the potential benefits of the complement system for different types of cancers [109,110,111,112,113]. The use of a C5aR1 inhibitor in combination with paclitaxel promotes interferon-γ (IFN-γ)-positive macrophage reprogramming increases the number and cytotoxicity of CXCR3+ effector and memory-CD8+T cells in the tumor microenvironment and enhances the efficacy and sensitivity of paclitaxel chemotherapy [114]. In glioblastoma, C5aR1 inhibitors in combination with temozolomide promote induced DNA damage and tumor cell apoptosis, thereby increasing sensitivity to chemotherapy [112]. Resistance to immunotherapy is a major problem that restricts anti-tumor therapy. Combined blocking of C5a and PD-L1 can affect the number of MDSCs in the TME and reduce their immunosuppressive function, thus reversing the depletion state of CD8+T cells, increasing the number of tumor-infiltrated CD8+T cells, and promoting the production of endogenous IL-10 to enhance the anti-tumor function [109]. Melanoma and colon cancer models have proved that dual inhibition of C5a and PD-L1 signaling pathways may contribute to synergistic effects in oncotherapy [111].

Currently, anti-C5aR1 in combination with anti-PD-1/PD-L1 is being studied in clinical trials for the treatment of patients with advanced solid tumors (NCT03665129). Adjuvant cancer therapy is utilizing monoclonal antibodies (mAbs) directed against complement regulatory proteins (CRPs), including CD46, CD55, and CD59 [115]. Sherbenou and colleagues reported that CD46 antibodies kill tumor cells by apoptosis while protecting benign cells [116]. Eculizumab, a humanized monoclonal antibody against C5, is the only one approved for human use and was approved to treat atypical hemolytic uremic syndrome and paroxysmal nocturnal hemoglobinuria [30]. However, eculizumab has never been tested, either in clinical trials or in preclinical models, on different types of cancer.

As far as we know, complement combination therapy has not yet been investigated in liver cancer. Therefore, in our review, the inhibition of the complement system can block the invasion, and migration of liver cancer cells and the formation of EMT. EMT can accelerate resistance to chemotherapy and radiotherapy [117]. Interference with specific EMT pathways in tumor cells is necessary to increase sensitivity to treatment. Tumor angiogenesis is known as a crucial step in tumor development and survival, thus making inhibition of angiogenesis one of the novel approaches to cancer treatment [118]. The inhibition of complement components also prevents vascular invasion of tumors. A combination of inhibiting the complement system and anti-angiogenic treatment can give a double blow to tumor vascular invasion and can impede tumor growth. We previously characterized the role of the complement system in responses of the TME and against tumor-associated antigens. Inhibition of the complement system could suppress the formation of immunosuppressive microenvironments by abrogating immune suppressor cells, including M2-like TAMs and TANs [71,79]. The impact of these characteristics on the efficacy of immunotherapy has a positive effect. In the same way, we realize that the combination of CSC-targeted treatment and conventional untargeted treatment can reduce chemotherapeutic resistance [119]. In the liver cancer model, the modulatory effect of complement on tumor immunity proves that it has a good effect on enhancing tumor immunotherapy. It prompts us to confirm the therapeutic potential of modulating complement system in liver cancer. The risks and benefits of combining complement-related therapies with antitumor therapies need further research.

## 5. Conclusions

Currently, research into the role of the different complement components at different clinical levels is just beginning to emerge. The complement system could have a significant role in tumor-associated immune responses, tumor cell biology, and stemness pathways. Though our review concentrates on the potential role of complement suppression in cancer treatment, killing tumor cells by the activation of complement components also represents a promising strategy. The findings of our review demonstrate that complement components and regulators might function as a biomarker and therapeutic target for liver cancer diagnosis and treatment. However, there does not exists a preclinical model that effectively recapitulates clinical liver cancer traits to clarify complement-related mechanisms concerned with liver cancer progression. A deeper understanding of the complement system, where tumors are concerned, is expected to create a new breakthrough in the treatment of liver cancer.

## Figures and Tables

**Table 1:** Summary of studies on the role of complement system in liver cancer.

Complement Component(s)	Role in Cancer	Experimental Setting	Mechanism	References
C5a and C5aR1	Pro-tumor	in vitro and vivo	Motivate Th-17 response in TAMs	[67]
C5a and C5aR1	Pro-tumor	in vivo	Promote M2 polarization of TAMs	[68]
C5aR1	Pro-tumor	in vitro and vivo	promote expression and secretion of IL-6	[69]
C3a and C3aR	Pro-tumor	in vitro and vivo	Generate iLDNs chemotaxis in liver tissue	[70]
C3	Pro-tumor	in vitro and vivo	Downregulate T cells and DC and upregulate MDSCs	[71,72]
C5a and C5aR1	Pro-tumor	in vitro	Enhance promotion of EMT	[73]
C3a and C3aR	Pro-tumor	in vitro	Enhance promotion of EMT	[74]
C5a and C5aR1	Pro-tumor	in vitro and vivo	Promote CTCs disseminating in distal organs	[75]
C5a and C5aR	Pro-tumor	in vitro and vivo	Enhance the expression of MMPs	[76]
C5aR1	Pro-tumor	in vitro and vivo	Stimulate JNK and ERK signal pathway	[69]
C3aR and C5aR1	Anti-tumor	in vitro	Generate apoptosis of cell cycle G0/G1 phase	[77]
CFR3	Pro-tumor	in vitro	Inhibit the PI3K/AKT/mTOR signal pathway	[78]
C1q	Pro-tumor	in vitro	Regulate β-Catenin pathway	[79]
C7 and CFH	Pro-tumor	in vitro and vivo	Generate formation of tumorsphere	[80]

TAMs: tumor-associated macrophages; iLDNs: low-density neutrophils; DC: dendritic cells; MDSCs: myeloid-derived suppressor cells; EMT: epithelial–mesenchymal transition; CTCs: circulating tumor cells; MMPs: matrix metalloproteinases.

## Data Availability

Not applicable.
